# An early interactive human coaching via a mobile application to improve quality of life in patients who underwent gastrectomy for gastric cancer: Design and protocol of a randomized controlled trial

**DOI:** 10.1371/journal.pone.0278370

**Published:** 2022-12-09

**Authors:** Hak Jin Kim, Hong Man Yoon, Keun Won Ryu, Young-Woo Kim, So Young Kim, Jin Myoung Oh, Gyung Ah Wie, Hyunyoung Ko, Jungeun Lee, Youngin Kim, Hyunsoon Cho, Bang Wool Eom

**Affiliations:** 1 Department of Cardiology, Center for Clinical Specialty, National Cancer Center, Goyang, Republic of Korea; 2 Center for Gastric Cancer, Research Institute & Hospital, National Cancer Center, Goyang, Republic of Korea; 3 Department of Clinical Nutrition, National Cancer Center, Goyang, Republic of Korea; 4 Research and Development Team, Noom Inc, Seoul, Republic of Korea; 5 Department of Cancer Control and Population Health, Graduate School of Cancer Science and Policy, National Cancer Center, Goyang, Korea; Prince Sattam Bin Abdulaziz University, College of Applied Medical Sciences, SAUDI ARABIA

## Abstract

**Background:**

After gastrectomy, patients may experience the postgastrectomy syndrome and face difficulties adapting to everyday diet. Recently, human health coaching via a mobile application (app) has been used for obese patients or patients with chronic diseases, with significant improvements in clinical outcomes. The aim of this study is to evaluate and compare the effects of human health coaching via a mobile app and conventional face-to-face counseling in patients who underwent gastrectomy for gastric cancers.

**Methods:**

This study is a single-institution, prospective randomized controlled trial comparing the mobile health and face-to-face counselling groups. After randomization, participants assigned to the mobile health coaching group will receive health coaching via a mobile app for 3 months after discharge, and the assigned coaches will provide personalized advice based on the self-recorded health data. Participants in the face-to-face group will have 1- and 3-months postoperative dietary consultations with a clinical dietitian. The primary endpoint is the food restriction score on the European Organization for Research and Treatment of Cancer Quality of Life Questionnaire-STO22, and secondary endpoints included all other quality of life scale scores and nutritional parameters. The calculated sample size is 180, and the outcomes will be measured until 1-year post-surgery.

**Significance:**

This study will show the efficacy of human health coaching via a mobile app on dietary adaptation in patients who underwent gastrectomy. A relational approach based on personal data and timely intervention using a mobile platform could reduce patients’ trial and error and improve quality of life.

**Trial registration:**

ClinicalTrials.gov, NCT04394585, Registered 19 May, 2020 –Retrospectively registered, http://clinicaltrials.gov/ct2/show/NCT040394585.

## Introduction

After gastrectomy, patients may experience the post-gastrectomy syndrome characterized by early satiety, abdominal fullness, indigestion, and dumping syndrome. During their hospital stay, patients are educated on a soft and regular dietary intake; however, it is up to them to adopt these dietary recommendations after discharge. The best way to relieve post-gastrectomy symptoms is to eat a small quantity of food more frequently. However, there are no detailed guidelines specifying the quantity of food to be consumed, the duration of dietary restriction, and the frequency of food consumption. In effect, it is impossible to develop a standardized dietary schedule for all patients because each patient is different in terms of eating habits, food preferences, and home environment. Therefore, personalized counseling and education are needed to help patients adapt to dietary modifications and reduce trial and error.

Recent studies have reported that nutritional counseling and support in the early stages of esophageal and gastric cancers improve health outcomes [1–3]. Early and intensive dietary consultation has been shown to reduce weight loss, improve physical function, and minimize nutritional status deterioration. One pilot study also showed improvements in nutritional status and weight loss through a telephone-based, early dietary counseling intervention [4]. Therefore, early interactive dietary counseling could assist patients in dietary adaptation, thereby improving patient quality of life (QoL).

Human health coaching is a patient-centered educational method that uses a relational approach to promote sense-making in health information and support goal-setting for health behavioral change [5]. Previous studies have highlighted specific goals can be attained through interpersonal relationships that enhance patient self-esteem and self-control. Health coaching has been shown to be effective in a variety of chronic diseases [6–8]. However, there were few studies to evaluate the effect of human health coaching in patients with gastric cancer. In this study, we aimed to evaluate whether human health coaching via a mobile application would improve quality of life in patients who underwent gastrectomy for gastric cancer.

## Materials and methods

### Mobile human health coaching programme

**Fig 1:**
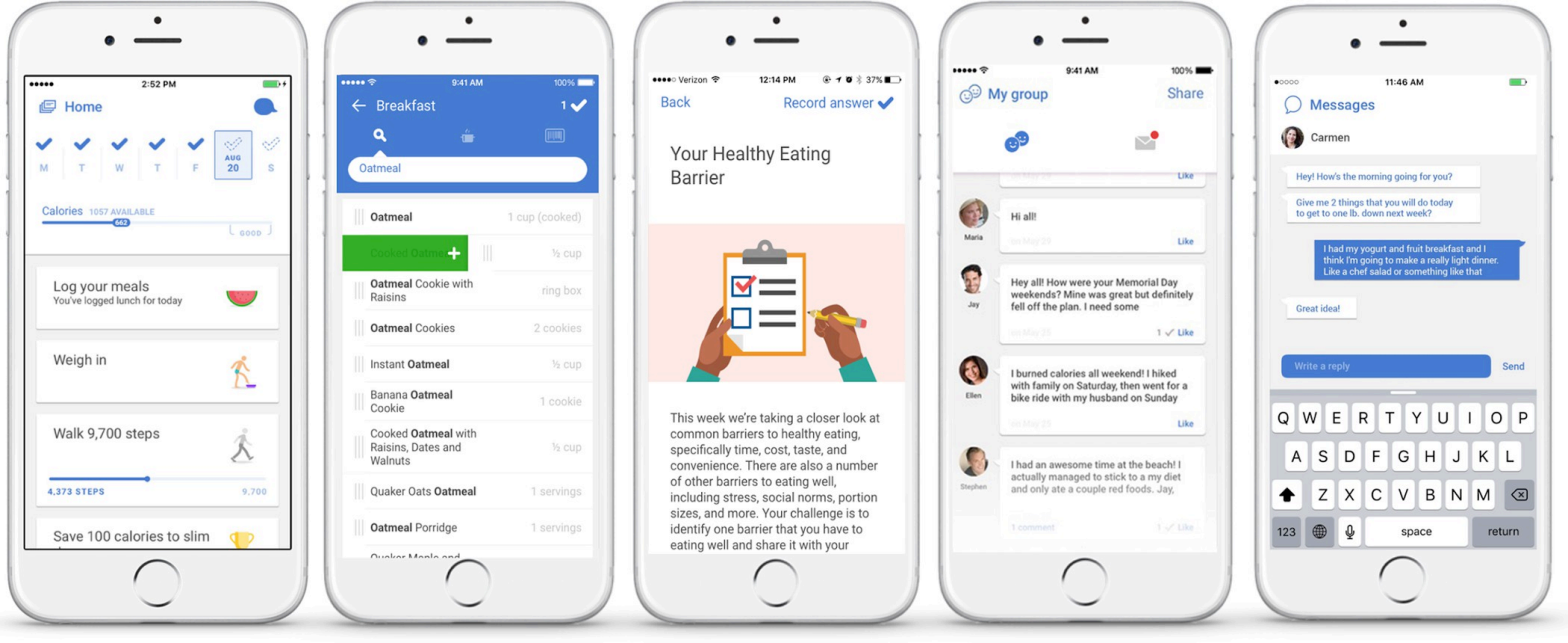
Screenshots of the Noom app for the participants (top) and screenshots of the dashboard for the coaches (bottom).

Noom Coach (Noom Inc.) is a commercialized mobile app that provides comprehensive human health coaching by connecting a participant with a coach (dietitian) for advice, instruction, and encouragement (Fig 1). Participants are asked to download the app on their smartphones, whereafter they are assigned to a coach following registration. Coaches encourage participants to log their daily food intake, daily exercise, and weekly body weight. Participants record the quantity of consumed food in the app, which automatically calculates and displays the total amount of energy consumed. Moreover, the app automatically measures and records the number of steps taken as participants walk with their smartphones. Informative in-app articles are provided twice or thrice weekly, and efforts are made to check whether the participants read the articles. Furthermore, using an in-app messenger system, coaches send messages to participants at least twice weekly to encourage them to follow the dietary and measurement guidelines and give individual advice based on each participant’s life-log. Participants are free to ask questions about nutritional and physical issues at any time via the app; moreover, the coaches answer the questions, as soon as possible, no later than the next morning. The study was conducted using a previous iteration of the Noom Coach program.

### Development of the human coaching program and results of a pilot study

From May to June 2019, the human coaching program for patients who underwent gastrectomy was developed by five physicians, three dietitians, two coordinator nurses, and three staff members of the Noom app, using conventional life-log input and communication systems between participants and coaches. However, new articles were written for patients who underwent gastrectomy. Twenty-six articles were written: 12 for nutritional or diet-related information, seven for post-gastrectomy syndrome, and seven for physical activities and mindfulness. Moreover, seven articles were independently written for patients with hypertension and included dietary approach for hypertension prevention, salt restriction, and physical activities. The articles have been revised several times to improve readability, and proper illustrations were added to enhance the reader’s understanding.

A pilot study was performed to evaluate the feasibility of the developed Noom Coach app for patients who underwent gastrectomy after getting approval from the institutional review board (IRB) of the National Cancer Center, Republic of Korea at 14 August, 2019 (IRB No. NCC2019-0210). Written informed consent will be obtained from all patients prior to recruitment. Primary outcomes were compliance and satisfaction after 3 months of using the Noom Coach app, and secondary outcomes were dropout rate, patient QoL assessed by the European Organization for Research and Treatment of Cancer Quality of Life Questionnaire Core 30 (EORTC QLQ-C30) and STO22, body weight, and body composition parameters (muscle and fat mass, and basal metabolism). The planned number of recruitments was 20.

Participants were enrolled at the National Cancer Center, Republic of Korea, between September and November 2019, and a complete enrolment was achieved (100%). Of the 20 participants, six dropped out due to advanced stages in the pathological report (n = 4) and patient withdrawal (n = 2). Thus, 14 participants were included in the analysis.

Seven of participants (50%) recorded their daily food intake and weekly body weight. However, the other seven participants were less compliant. Eight participants read more than 75% of the articles during the 3 months. All participants (100%) admitted the efficacy of the Noom Coach app in diet adaptation and were satisfied with the use of the app (12 strongly agreed and two weakly agreed). Compared to preoperative values, patient QoL significantly worsened 1-month postoperatively, including physical, role, and social functions, as well as fatigue, pain, appetite loss, diarrhea, dysphagia, eating restriction, anxiety, dry mouth, taste, and body image. The QoL deterioration lasted up to 3 months in social function, fatigue, diarrhea, eating restriction, and body image. In particular, eating restriction was the most significantly altered parameter. Muscle mass and basal metabolism significantly decreased 1 and 3 months postoperatively; however, there were no significant differences in fat mass, body fat percentage, and body mass index.

Based on this pilot study, we designed a prospective randomized controlled study comparing the effect of human health coaching via a mobile app and face-to-face counselling on QoL in patients who underwent gastrectomy for gastric cancer. The mobile app, Noom Coach will be used for human health coaching.

### Study design

**Fig 2:**
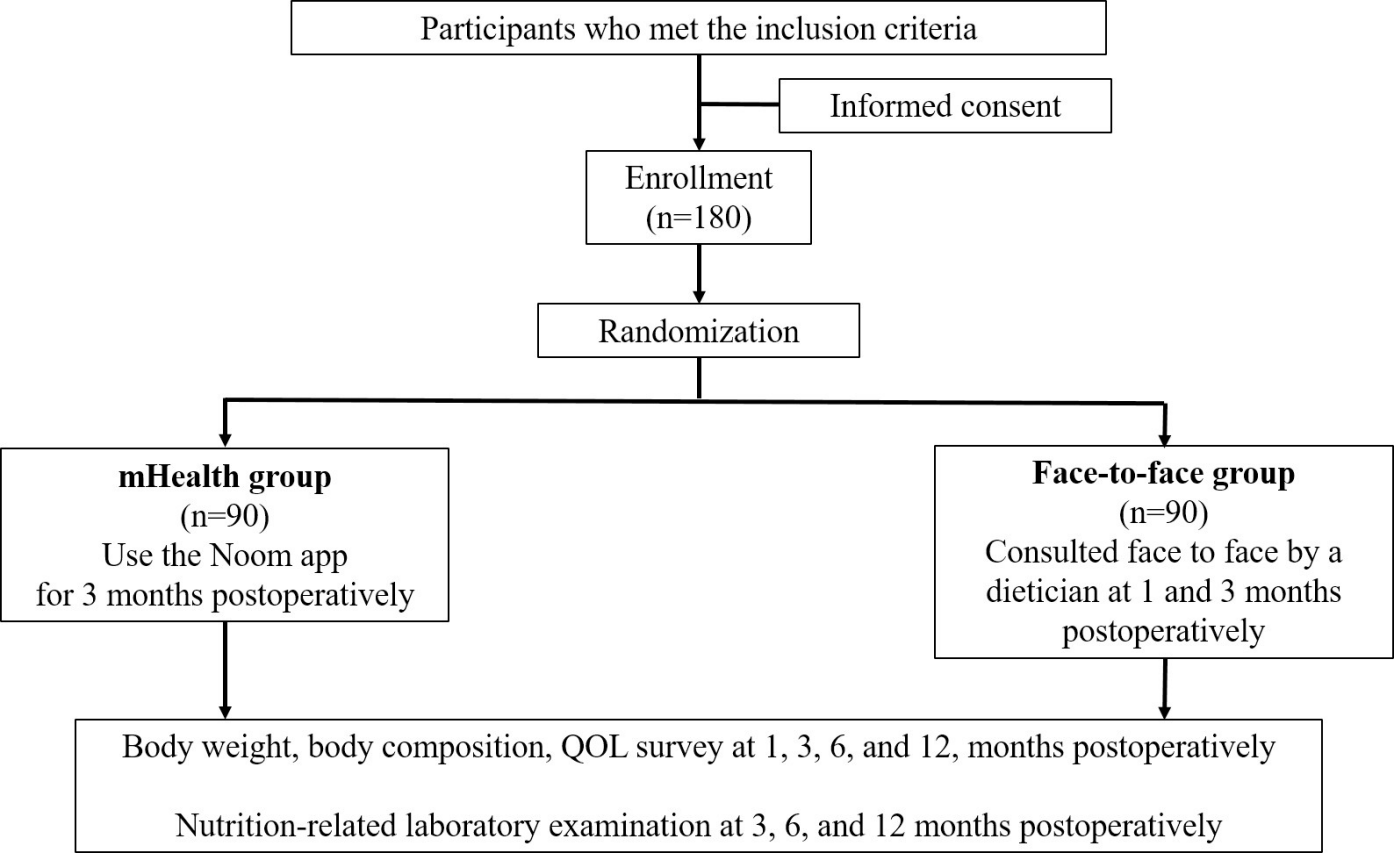
Study flow.

This study is an investigator-initiated, single-center, prospective, randomized controlled trial to evaluate the effect of early interactive human coaching via a mobile app on QoL compared with face-to-face counseling in patients who underwent gastrectomy for gastric cancer (Fig 2). The IRB of the National Cancer Center, Republic of Korea has approved this study at 13 June, 2019 (IRB No. NCC2019-0137) and written informed consent will be obtained from all patients prior to recruitment. The first patient was enrolled at 11 May, 2020 and the estimated date for completion of recruitment is 31 December, 2021. The authors confirm that all ongoing trials for this intervention are registered, and this study has been registered in clinicaltrials.gov (NCT04394585). The registration date was 8 days later than the first participant enrolment because we overlooked that it is important to enrol a participant to a trial after the trial was registered.

### Participants and eligibility criteria

Participants will be recruited at the outpatient clinic after preoperative evaluation for gastric cancer. The inclusion criteria are: (1) patients aged at least 19 years, (2) diagnosed with clinical stage I gastric cancer on preoperative esophagogastroduodenoscopy and computed tomography, (3) scheduled to undergo distal or total gastrectomy, (4) able to access the mobile app via a smart phone or live with the help of a caregiver, and (5) cognitively capable of communicating with an assigned coach. The dropout criteria are: (1) patients whose planned gastrectomy is eventually not performed, (2) those with a hospital stay of more than 3 weeks due to postoperative complications, and (3) those who request withdrawal from the study.

### Randomization

The Clinical Research Coordination Center of the National Cancer Center, Republic of Korea will coordinate this study and handle the data using a web-based clinical trial management system (myTrial, Bethesda Soft Co., Ltd., Seoul, Republic of Korea: http://www.mytrial.co.kr). Patients scheduled for gastrectomy will be consecutively screened for eligibility, and a surgeon will explain this study to both the patients and their caregivers. Patients who agree to participate will sign a consent form. Thereafter, they will be registered in the management system by a coordinator and randomly allocated to either the mHealth or the face-to-face group following a 1:1 ratio. The random block size permutation method will be used to generate the initial randomization sequence, and the randomization will be stratified based on the extent of gastrectomy (distal vs. total gastrectomy).

### Intervention and conventional support

All patients will undergo curative radical gastrectomy according to clinical guidelines [9,10]. Distal gastrectomy with D1+ (Nos. 1, 3, 4sb, 4d, 5, 6, 7, 8a, and 9) or D2 (Nos. 1, 3, 4sb, 4d, 5, 6, 7, 8a, 9, 11p, and 12a) lymph node dissection will be performed for tumors located in the middle or lower one-third of the stomach. For tumors located at the upper one-third or wide spread tumors, total gastrectomy with D1+ (Nos. 1–7, 8a, 9, and 11p) or D2 (Nos. 1–7, 8a, 9, 10, 11p, 11d, and 12a) lymph node dissection will be performed. All patients will receive general postoperative management during their hospital stay.

Prior to discharge, patients assigned to the mHealth group will download the Noom app on their smartphones and will be instructed on how to use the app. Participants will receive nutritional advice and counseling from an assigned dietitian via the Noom app for 3 months postoperatively.

Patients assigned to the face-to-face group will have a face-to-face consultation with a dietitian during their at 1 month and 3 months postoperatively follow-up hospital visits.

### Measurements

The primary outcome measure is the eating restriction scale score on the EORTC QLQ-STO22 at postoperative 1 month. The EORTC QLQ-STO22 is a 22-item gastric cancer-specific questionnaire composed of nine symptom scales (dysphasia, pain, reflux symptoms, eating restrictions, anxiety, dry mouth, taste, body image, and hair loss) [11]. Each scale is represented by a score ranging from 0 to 100. A higher score indicates more severe symptoms and poorer QoL.

**Fig 3:**
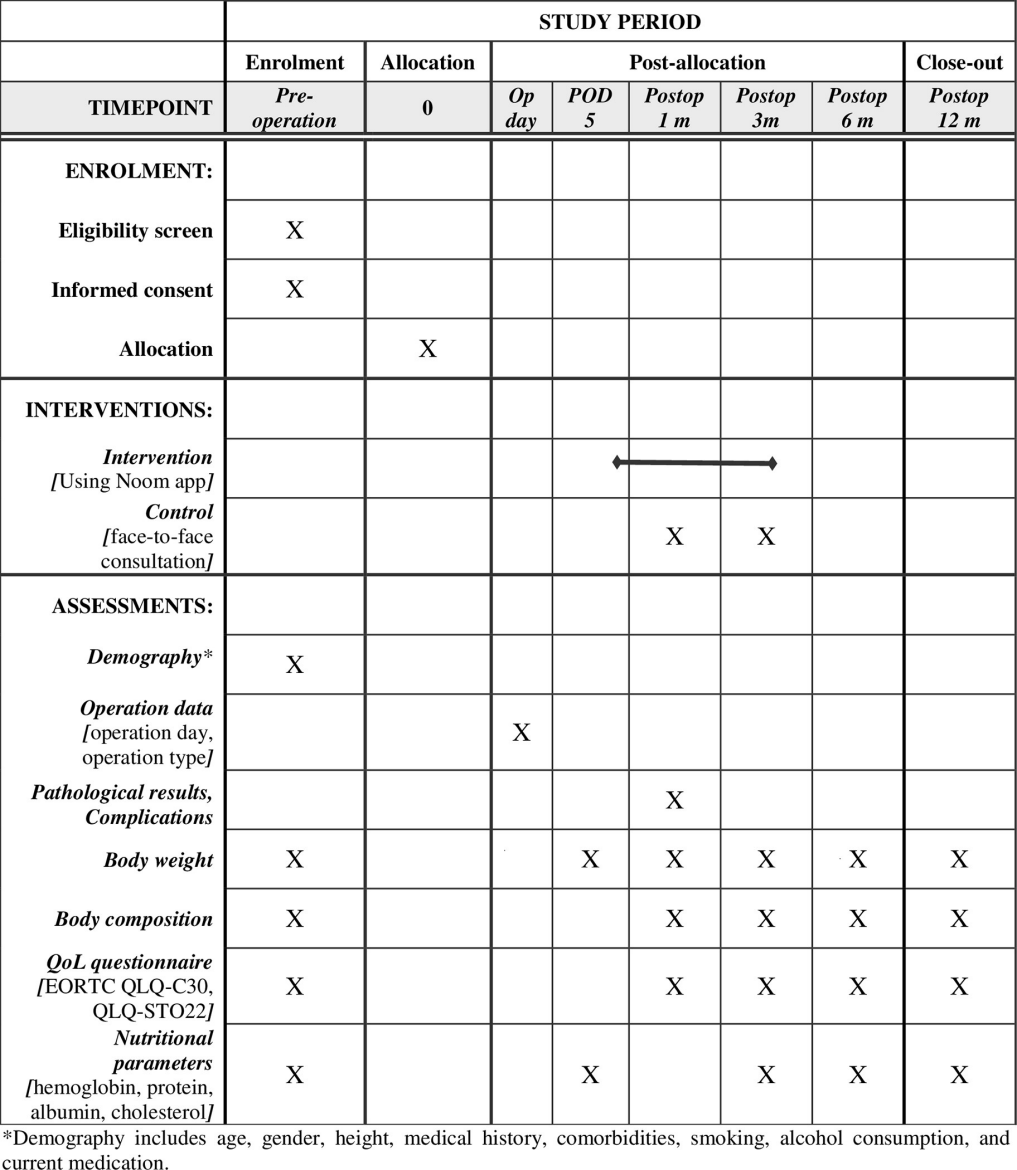
Schedule of enrolment, intervention, and assessments.

The secondary outcomes are QoL changes over time assessed by the EORTC QLQ-C30 and the EORTC QLQ-STO22. The EORTC QLQ-C30 is a 30-item questionnaire evaluating the general QoL of patients with cancer [12]. It consists of six functions (physical, role, emotional, cognitive, and social function), nine symptoms (fatigue, nausea and vomiting, pain, dyspnea, insomnia, appetite loss, constipation, diarrhea, and financial difficulties), and the global health status. A high score for the functional and global health status scales indicates a good functional capacity and QoL, whereas a high score on the symptom scale indicates severe symptoms and poor QoL [13]. Participants will complete the questionnaires preoperatively and at 1, 3, 6, and 12 months postoperatively (Fig 3).

Other secondary outcomes are nutritional parameters, including body weight, body mass index, body composition (muscle mass, fat mass, body fat percentage, basal metabolism, etc.), and nutrition-related laboratory values (hemoglobin, serum protein, albumin, and total cholesterol). Body weight will be recorded daily via the app and will be measured on postoperative day 5 and on every outpatient follow-up visit. Body composition will be estimated using a bioelectrical impedance analyser (InBody, Seoul, Korea), and blood samples will be collected for laboratory analyses during the outpatient follow-up visits to the clinic.

Demographic and baseline clinical data will be collected including age, gender, height, medical history, comorbidities, smoking, alcohol consumption, and current medication. Operation type and pathological results including tumor stage will also be collected.

### Sample size

The sample size was calculated based on the results of our pilot study and previous QoL studies which evaluated EORTC QLQ-STO22. In this study, the primary outcome is the eating restriction scale score of the EORTC QLQ-STO22, which was 30 points at postoperative 1 month in the pilot study. We also assume an intergroup difference of 5 points and standard deviation of 10 based on the results of previous QoL studies, yielding an effect size of 0.5 [14–17]. Given a type I error of 0.05, 80% statistical power, and 1:1 enrolment ratio, 126 participants were calculated using a 2-tailed t-test. Allowing for an 30% of dropout rate based on the results of the pilot study, we planned to recruit 180 participants (90 in each group) for this study.

### Statistical analysis

In descriptive statistics, continuous variables will be shown as the means ± standard deviations or the medians with interquartile ranges, and the categorical variables will be presented as proportions. Intergroup differences will be tested using a t-test or the Wilcoxon rank-sum test for continuous variables and the chi-square test or Fisher’s exact test for categorical variables. In QoL analysis, each subscale or item will be presented as the median and interquartile range. Nonparametric statistics (i.e., the Wilcoxon rank-sum test) will be used to evaluate the statistical significance of QoL scores that do not follow a normal distribution.

For the analysis of QoL changes and nutritional outcomes over time, linear mixed models will be used for intergroup comparisons. Fixed effects include groups, time, and the interaction between group and time, and the differences in QoL scores and nutritional values between the groups and changes in QoL scores and values between baseline and follow-up will be estimated. Before analyses, the normality of the distribution of the QoL scores and nutritional parameters will be determined. Normality assumptions of the linear mixed models will be checked. If they don’t have a normal distribution, generalized estimating equation can be applied for the analyses. If the normality assumption is not met, the analysis will be performed by model fitting after suitable transformations for the responses or by exploring the family of models that accommodates the distribution of the data. Graphs will be constructed in which the mean values over time on the subscales of the QoL scores and nutritional parameters will be presented.

Data analyses will be conducted using SAS 9.4 (SAS Institute Inc., Cary, NC, USA) and R software, and P-values less than 0.05 will be considered significant.

## Discussion

Dietary adaptation and return to normal life are major challenges for patients who underwent gastrectomy. This study will evaluate and compare the effect of human health coaching via a mobile app and face-to-face counselling on QoL improvement in patients who underwent gastrectomy. This is the first study focusing on diet adaptation after gastrectomy for gastric cancer using a mobile platform and would provide the evidence of the effect of mobile human health coaching on diet adaptation.

Various nutritional interventions have been used in clinical trials, the most common of which is face-to-face counseling by a dietitian. Individualized dietary counseling along with nutritional education has shown a positive effect on QoL with improved food intake and nutritional status [18–21]. Telephone calls are also commonly used in nutritional interventions, with proven benefits on clinical outcomes, such as weight management and QoL improvement [22–24]. Some studies used text messages or mail, and reported that participants achieved significant improvement in weight control or osteoporosis management [24–28]. Recently, nutritional and lifestyle interventions via mobile app platforms have been introduced and applied to weight control or diabetes management [29–31]. Using this mobile platform, participants can easily record their health data, such as body weight, diet diary, and daily exercise, and can be more compliant with self-monitoring. Health coaches can provide personalized nutritional advice based on recorded personal data and timely responses to participant questions. Moreover, health education, goal setting, and encouragement to complete tasks can also be made available by health coaches. Therefore, this health coaching method via a mobile app platform is highly effective and advantageous.

There are very few studies on nutritional interventions using mobile platforms in patients with gastric cancer. A study protocol of a randomized controlled trial evaluating the effect of early intervention delivered via a mobile app on QoL in people with upper gastrointestinal (esophageal, gastric, and pancreatic) cancer was reported [32]. The aforementioned study recruits not only patients who will undergo surgery but also those who will receive chemotherapy or radiotherapy, and nutritional intervention could be included to reduce the side effects of chemotherapy or radiotherapy and manage the symptoms of gastrointestinal cancer. Another difference is the main outcomes. The primary outcome of the study is quality-adjusted life years lived using the EuroQol-5 Dimension-5 Level and secondary outcomes are 12-month survival and body weight. The life expectancy of patients with advanced disease is not so long and survival outcome can be measured in the aforementioned study. However, in our study, we will mostly recruit participants with an early-stage gastric cancer for which curative resection will be performed. Therefore, our study will focus on diet adaptation after gastrectomy, and we will compare short-term QoL and nutritional parameters.

This study was designed based on pilot study. Before the pilot study, it was difficult to expect how many participants actively use the mobile app and enjoy the communication. In the Republic of Korea, approximately half of the number of patients diagnosed with gastric cancer are aged 65 years or older, and they may have difficulties using the mobile app [33]. Moreover, it was uncertain how many participants will drop out from the study due to dissatisfaction with the app. Through the pilot study, we found that half number of participants actively used the app to report their life-log. Only two of the 20 patients withdrew from the study. The 1-month postoperative QoL was worst, however, it was partially ameliorated thereafter. Based on these findings, we formulated a hypothesis that the 1-month postoperative eating restriction will be better in the mobile app group than in the face-to-face group. The sample size was also calculated by referring to the dropout rate in the pilot study.

Our team including physicians, clinical coordinators, dietitians, and coaches of Noom app, have regularly held meetings since the start of this study. Coaches share patients’ needs and problems, and physicians give comments on how to reply to patients on pertinent issues. With the efforts of our team, we assumed that human coaching via mobile apps could better respond to patients’ needs and help patients return to normal life quickly.

## Conclusions

We expect this trial can provide the evidence for the effectiveness of human health coaching via a mobile app on dietary adaptation in patients who underwent gastrectomy for gastric cancer. A relational approach based on personal data and timely intervention using a mobile platform can reduce patients’ trial and error and improve QoL. Moreover, this human health coaching via a mobile app can be applied to patients with other cancers or diseases who need dietary or life-style coaching. Further well-designed studies on the effectiveness of human coaching via a mobile app are necessary in the future.

## Supporting information

S1 File (DOCX)

S2 File (PDF)
