# Supplementary material for: An early interactive human coaching via a mobile application to improve quality of life in patients who underwent gastrectomy for gastric cancer: Design and protocol of a randomized controlled trial
Source: PLoS One. 2022 Dec 9;17(12):e0278370. doi: 10.1371/journal.pone.0278370 (PMC9733875; doi:10.1371/journal.pone.0278370)
Supplement: S1 File — (DOCX) [file pone.0278370.s001.docx]

Research proposal

Study title: A study evaluating effect of smart phone app-based human coaching program on QOL in patients who underwent gastrectomy for stage I gastric cancer

Version: 1.1 Date of version: 2019-05-28

Principle investigator: Center for Gastric Cancer Bang Wool Eom

Co-investigator:

Department of Cardiology Hak Jin Kim

Center for Gastric Cancer Young-Woo Kim

Center for Gastric Cancer Keun Won Ryu

Center for Gastric Cancer Hong Man Yoon

Department of Clinical Nutrition So Young Kim

Department of Clinical Nutrition Jin Myoung Oh

Department of Clinical Nutrition Gyung Ah Wie

Graduate School of Cancer Science and Policy Hyun Soon Cho

Research and Development team, Noom Inc Hyunyoung Ko

Research and Development team, Noom Inc Jungeun Lee

Research and Development team, Noom Inc Youngin Kim

NATIONAL CANCER CENTER

323 Ilsan-ro, Ilsandong-gu, Goyang-si

Gyeonggi-do, 10408, Republic of Korea

Absract

| **Study title** | A study evaluating effect of smart phone app-based human coaching program on QOL in patients who underwent gastrectomy for stage I gastric cancer | | | | |
| --- | --- | --- | --- | --- | --- |
| **Investigators of National Cancer Center** | Role | Affiliation | Name | Participation in the consent process  Description of the study | Participation in the consent process  Signing the agreement |
|  | PI | Center for Gastric Cancer | BWE | Present | Presenㅅ |
|  | Coinvestigator | Department of Cardiology | HJK | Present | Present |
|  | Coinvestigator | Center for Gastric Cancer | YWK | Present | Present |
|  | Coinvestigator | Center for Gastric Cancer | KWR | Present | Present |
|  | Coinvestigator | Center for Gastric Cancer | HMY | Present | Present |
|  | Coinvestigator | Department of Clinical Nutrition | SYK | Absent | Absent |
|  | Coinvestigator | Department of Clinical Nutrition | JMO | Absent | Absent |
|  | Coinvestigator | Department of Clinical Nutrition | GAW | Absent | Absent |
|  | Coinvestigator | Graduate School of Cancer Science and Policy | HSC | Absent | Absent |
| **Joint research institute** | Noom Inc/ Hyunyoung Ko  Noom Inc/ Jungeun Lee  Noom Inc/ Youngin Kim | | | | |
| **Purpose** | ○ **Primary purpose:**  - Analysis of the effect of using a smartphone app-based human coaching program on the patient's symptoms and quality of life in patients who underwent gastrectomy for stage 1 gastric cancer  - Comparison of food restriction scale scores of EORTC QLQ STO22 at 3 months after surgery in patients who used smartphone app and patients who received nutrition counseling  ○ **Secondary purpose:**  - Comparison of food restriction scale scores of EORTC QLQ C30 and STO22 at 6 months after surgery  - Comparison of food restriction scale scores of EORTC QLQ C30 and STO22 at 12 months after surgery  - Meal volume, biomarkers (BMI, muscle mass, etc.), blood test results | | | | |
| **Background** | - Patients who underwent gastrectomy suffer from symptoms such as abdominal pain, diarrhea, nausea/vomiting, increased pulse, weakness, and hypoglycemia (gastric resection syndrome) after a meal due to the resection of the pylorus that controls the speed of food movement between the stomach and small intestine. Symptoms are especially severe when the patient rushes to eat food, eats a large amount at once, and eats high-sugar foods.  - During the hospitalization period, the patient begins with Mieum and proceeds with a small amount of porridge, but after discharge, he suffers trial and error because he does not know how to proceed in his daily life. In addition, after being diagnosed as a cancer patient, people around them listen to unverified stories and eat mainly with certain foods, or they may not be able to consume enough nutrition due to restrictions on certain foods. Sometimes suffered.  - Currently, a smartphone app (Noom) that provides a 1:1 personalized coaching service is being used among the general public to keep a daily meal diary and calculate meal calories for weight loss. Noom not only makes it easy for users to enter the food eaten during the day based on the food database, but also helps the coach in charge check daily to keep the dietary control.  - If a gastric cancer patient uses Noom in the process of nutritional adaptation after surgery, coaching can be performed at an appropriate time according to the patient's situation. As a result, patients and their caregivers are expected to reduce trial and error related to dietary adaptation and reduce gastrectomy syndrome. | | | | |
| Results of  previous study | □ Absent ☑ Present  - In the past literature, the results that weight control and metabolic syndrome control were significantly improved when using a smartphone app were published in several papers.  - There have also been studies showing that breast cancer survivors, which are highly related to obesity, lose weight, increase vegetable intake, and increase exercise volume when nutritional control using a smartphone app is used.  - Regarding stomach cancer, a study on improving lung capacity with a mobile self-monitoring tool has been published, and a protocol has been introduced for studies related to nutrition or diet.  **< Pilot study>**  ○ Title: Investigation of the effectiveness of QOL improvement using a smartphone app-based human coaching program in patients who underwent gastrectomy for stage 1 gastric cancer.  ○ Research content: Patients who underwent gastrectomy for stage 1 gastric cancer receive individual coaching using a smartphone app on health management methods after gastrectomy such as weight, diet, and exercise for 3 months from the date of discharge. EORTC QLQ questionnaire was answered at 1 and 3 months after gastrectomy, and satisfaction with human coaching was surveyed. The patient's body count and nutritional results on blood tests are also collected.  ○ Patient enrollment  -Enrollment period: 2019.9.19. ~ 11.6  -Number of enrolled patients (Actual/Planned): 20/20 (100%)  -Number of drop-out: 6/20 (30%)  -Reason of drop-out:  -Diagnosed as stage 2 or higher in the final pathology examination (n=4)  -Refusal (n=2)  ○ Results  1) Eating restriction score of EORTC QLQ-STO22  -Preoperative, median value: 6.7 (IQR 6.7, 13.3)  -Postoperative 1 month, median value: 30.0 (IQR 13.3, 56.7)  -Postoperative 3 month, median value: 20.0 (IQR 3.3, 26.7)  2) Satisfaction:  : The use of the app helped adapt to the diet and the overall satisfaction was very high.  -Ease of use of the app – Strongly yes (9), yes (3), no (1)  -Ease of understanding of the information provided - Strongly yes (8), yes (4)  -Usability of using the app - Strongly yes (12), yes (1)  -Appropriateness of time spent in app - Strongly yes (8), yes (5)  -Overall satisfaction: Strongly yes (11), yes (2) | | | | |
| **Study overview** | ○ Study design: single center, prospective, randomization  ○ Research participation period: One year after gastrectomy for early gastric cancer  ○ Data collection period: IRB approval day – 2021. 12. 31  ○ Overall study period: IRB approval day – 2022. 6. 30. | | | | |
| **Data collection items and**  **Clinical test items** | ○ Demography: age, sex, name initial  ○ Anthropometric information: height, body weight, body component such as muscle mass measured with a body composition analyzer  ○ Past medical history: past medical history, smoking, alcohol, concomitant medications  ○ Diagnostic, pathological data: operating day, operation method, pathological results of gastric cancer, postoperative complications  ○ Laboratory findings: Hemoglobin, Protein, Albumin, Cholesterol  ○ Questionnaires: EORTC QLQ C30, STO22 | | | | |
| **Research participation condition** | ☑inpatient ☑outpatient □ local residents □ others | | | | |
| **Inclusion criteria** | 1. Patients over 19 years of age  2. Patients diagnosed with stage 1 gastric cancer before surgery  (Early gastric cancer at preoperative endoscopy or stage I at CT scan)  3, Patients scheduled to undergo distal or total gastrectomy  4. Patients who are able to use the smartphone app or a care giver living with him can use the app to actively intervene in the patient's life.  5. Patients who have written research consent | | | | |
| **Exclusion criteria** | 1. Vulnerable patients (pregnancy, a person who lacks the ability to speak)   2. Patients who cannot use the smartphone app or the active intervention of a care-giver is difficult | | | | |
| **Drop-out criteria** | 1. Patients who have not undergone distal gastrectomy or total gastrectomy 2. The pathology test result is stage II or higher after gastrectomy 3. Patients who need adjuvant chemotherapy after gastrectomy   4. The hospital stay exceeds 3 weeks due to complications  5. Patients request withdrawal of participation  6. Patients having difficulty in collecting data suitable for the purpose of the study because they have not visited at all 1, 3, 6, and 12 months | | | | |
| **Study method** | - Patients who are scheduled to undergo gastrectomy for stage 1 gastric cancer are randomly assigned either to the mHealth group or the face-to-face counseling group. Patients in the mHealth group (N=90) will use mobile application for 3 months after discharge and those who in the face-to-face counseling group (N=90) will receive general nutrition counseling at 1 month and 3 months after gastrectomy.  - QOL, body component, and nutritional outcomes will be compared between the two groups.  - When obtaining the consent form, the mHealth group will be provided with a simple noom instruction manual and a noom meal record guide, and will be informed of how to use it.  - In addition, individual coaching will be given through articles and messengers on how to manage post-gastrectomy such as weight, diet, and exercise on the mobile app for 3 months from the discharge date.  - Survey response on quality of life at outpatient visits 1, 3, 6, 12 months after surgery  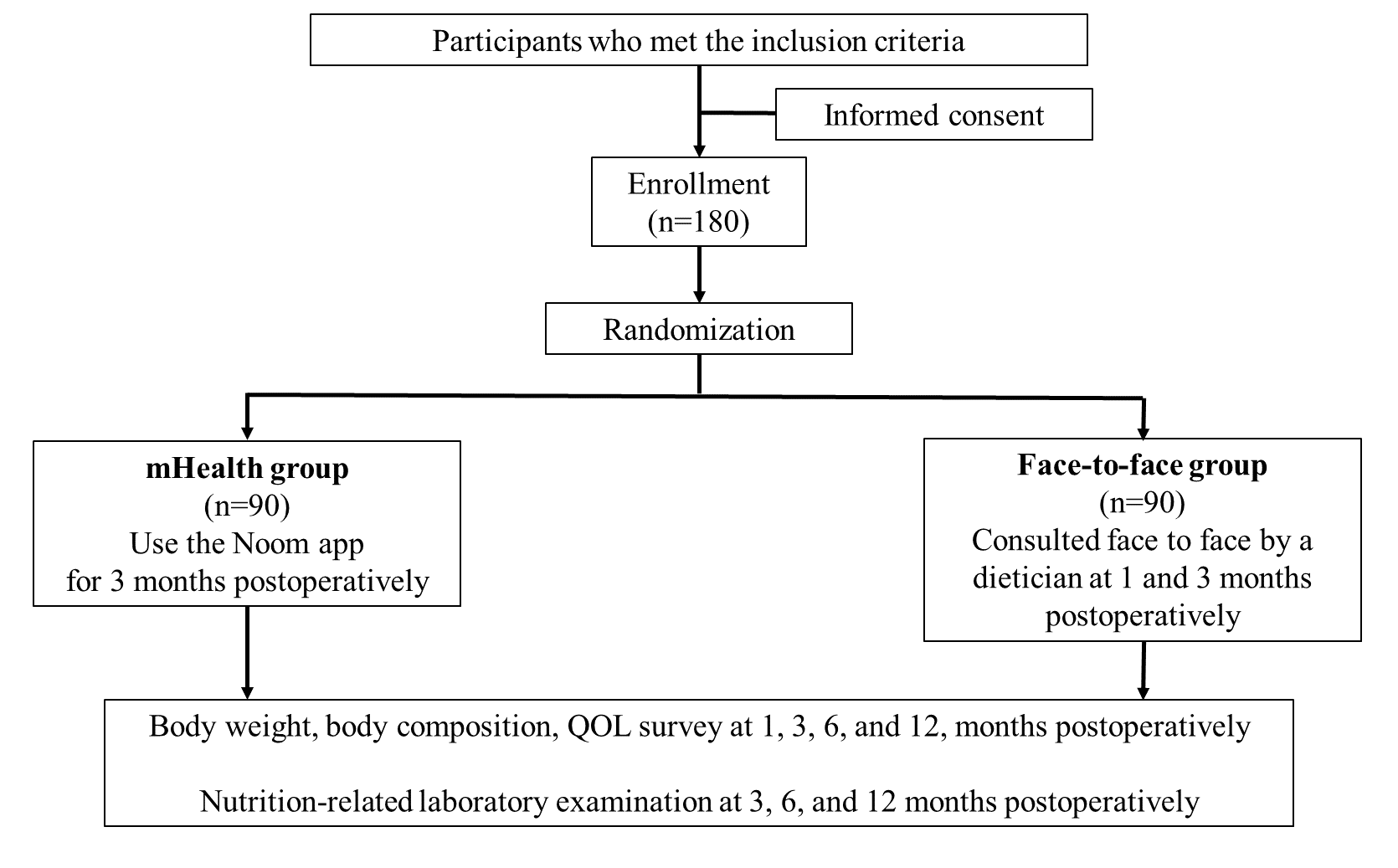  * The allowable date of visit at 1, 3, 6, and 12 months after surgery is 1 month before and after. | | | | |
| **Intervention method** | **- mHealth group:** After being discharged from hospital, patients will receive coaching using a smartphone app for 3 months. When the patient is discharged from the hospital, he downloads the Noom app on his or her mobile phone, registers, and then uses the Noom program. In addition, a personal coach is assigned to provide appropriate coaching to the patient on the app.  **-Face-to-Face group:**　Patients will receive individual nutrition counseling from a clinical nutritionist at the hospital at 1 month and 3 months after surgery. | | | | |
| **Evaluation** | Between the mHealth and face-to-face groups  - Comparison of scores of EORTC QLQ-C30 and STO22 at 1, 3, 6, and 12 months postoperatively.  - Comparison of meal amount at 1, 3, 6, and 12 months postoperatively.  - Comparison of body component (BMI, muscle mass, etc) at 1, 3, 6, and 12 months postoperatively.  - Comparison of nutritional outcomes (hemoglobin, albumin, protein, cholesterol) at 1, 3, 6, and 12 months postoperatively | | | | |
| **Number of research subjects** | - In the results of the pilot study, the eating restriction score of EORTC QLQ-STO22 that was answered at 1 month postoperatively was 30 points. The drop-out rate of the entire study was 30%. Based on this, the following hypothesis was established.  - Among the EORTC STO22 items that patients in the mHealth group responded to 1 month after surgery, the score for the eating restriction item was expected to be 30±10 (mean±standard deviation). The item score for the control group was expected to be 35 points.  - alpha value: 0.05  - power: 80%  → sample size was calculated as 65 cases in each group.  → Considering 30% of drop-out rate, a total of 180 cases (90 cases in each group) is needed.  - If drop-outs are more than expected, the number of sample size can be adjusted through statistical advice. | | | | |
| **Statistical consideration** | **1. Randomization**  - Randomization generally performs permutated mixed block randomization, and is generated in a 1:1 ratio using 2 and 4 mixed block blocks. Using the nQuery Program, a randomly assigned table is created according to the stratification factor and notified through a web-based clinical research management system.  - Stratification factors: extent of gastrectomy (distal vs. total gastrectomy)  2. **Statistical analyses**  - Patient's clinical characteristics are expressed as mean ± standard deviation or ratio using descriptive statistics.  - Significance was evaluated by t-test for continuous variables and Chi-square test for discontinuous variables.  - For the values ​​repeatedly measured at time intervals, the difference between the two groups was compared using a mixed effect model. | | | | |
| **Specimen** | ☑Not applicable | | | | |
| **Plan for secondary use of collected data** | □ Not applicable  □ Use of personal information including personally identifiable information  ☑ Use of personal information except personally identifiable information  □Use of specimen | | | | |
| **Monitoring plan** | ○ This study is conducted through the use of smartphone apps and questionnaires of the subjects, and safety issues are not applicable.  ○ Adverse reactions are not collected because there are no adverse reactions in the use of smartphone apps or surveys. | | | | |
| **Ethical consideration** | ○ Protection measures for vulnerable research subjects ⍌ Not applicable  ○ How to use personal information including personally identifiable information  - Personally identifiable information: hospital registration number, name, telephone number, birth year  - Personal information: age, sex, height, weight, past medical history, smoking status, alcohol consumption, concomitant medication use, operation date, surgical method, gastric cancer pathology result, postoperative complications, BMI, body composition analysis and body measurement results, Hb, Protein , Albumin, Cholesterol  - Purpose of using personal information, including personally identifiable information:  Search of medical record, smartphone app use and human coaching  - Use period of personal information including personally identifiable information:  (1) Stored for 3 years after the end of the study  (2) When a participant requests deletion of personal information, it will be deleted immediately  ○ Personal Information Protection Plan  - Subjects have the right to refuse consent to collection and use of personal information, and participation in this study is restricted if consent is rejected (Article 15 of the Personal Information Protection Act, collection and use of personal information), and minimum personal information necessary for the purpose of the study (Article 16 of the Personal Information Protection Act, Restriction on Collection of Personal Information). Records related to personal information will be kept for 3 years from the time the research is completed in accordance with Article 15 of the Enforcement Rule of the 「Life Ethics and Safety Act」. This past document will be destroyed (Article 21 of the Personal Information Protection Act, destruction of personal information).  - Disposal plan for data after research is completed: Research data including personal information will be disposed of as follows in accordance with Article 16 of the Enforcement Decree of the Personal Information Protection Act after the storage period.  (1) Information stored in the form of electronic files is permanently deleted in a way that cannot be restored through initialization or overwriting to prevent data from being restored.  (2) Written printed materials are completely destroyed through a shredder.  - The PI oversees the disposal process and ensures that the material has been completely destroyed.  - Protection methods for research subjects and to ensure confidentiality of personal information  (1) Only the minimum personal information necessary for the purpose of this study will be collected, and the collected personal identification information and personal information will not be leaked or shared with others other than the responsible researcher and researcher  (2) Data will be managed by assigning a research ID, and the information collected for research will be stored as an electronic file with a password in a locked laboratory. Only PI and researchers can access the research file.  (3) It will be permanently deleted by PI after storage for 3 years after the end of the study.  ○ Damage compensation plan: Not applicable  ○ Support matters for research subjects: □Absent ☑Present  - Free use of smartphone app (Noom) for 3 months  - Nutrition education at 1 month and 3 months after surgery  ○ Additional costs incurred when a research subject participates in the research:  ☑Absent □Present  ○ Data management plan  (1) Data is managed by assigning a research ID, and information collected for research is stored in an electronic file with a password in a locked laboratory.  (2) Research files can only be accessed by PI and researchers, and collected documents are kept in a lockable drawer to limit access.  (3) The collected data is stored for 3 years after the end of the study and then permanently disposed of by the lead researcher.  (4) The identifier code associated with the subject's personal information is restricted by PI by storing it in a lockable chest of drawers. | | | | |

※ “Personal identification information” refers to information that can identify an individual, such as the name and resident registration number of a research subject.

※“Personal information” refers to personal information, such as personal identification information, genetic information, or health information.

**1. Background**

**(1) Changes in the diet of patients after gastrectomy**

- Patients who underwent gastrectomy experience the following anatomical changes.


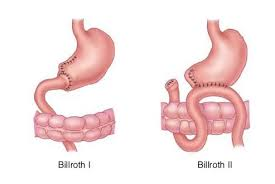
- Patients suffer from gastrectomy syndrome due to resection of the pylorus, which regulates the speed of food movement between the stomach and small intestine. Gastric resection syndrome includes abdominal pain, diarrhea, nausea/vomiting, increased pulse, weakness, and hypoglycemia that occur after eating.

- In particular, when the patient rushes to eat or eats a large amount of food, and eats high-sugar foods, gastrectomy syndrome can be severely experienced.

**(2) Dietary adaptation of the patient**

- Patients receive nutritional counseling during the hospitalization period, but when they return to their daily life after discharge, there is no separate education on how to eat and how to adjust, resulting in various trials and errors.

- In particular, after being diagnosed as a cancer patient, there are cases where people around us hear unverified stories and eat mainly with certain foods, or they may not be able to consume enough nutrition due to restrictions on certain foods.

- They are also not well aware of postgastrectomy syndrome, so they constantly experience discomfort after eating.

- This postgastrectomy syndrome is most severe in the early postoperative period and gradually relieves after dietary adaptation.

(3) **Diet management using a smartphone app**


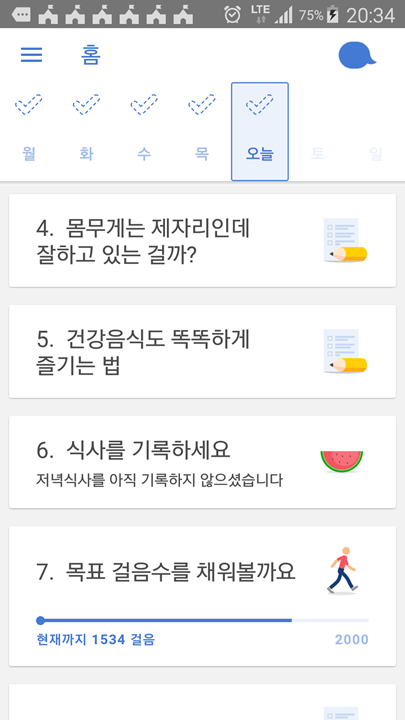
- Currently, for weight loss, a smartphone app (Noom) is being used that keeps a daily meal diary, calculates meal calories, and provides 1:1 customized coaching service.

- Noom not only makes it easy for users to enter the food eaten during the day based on the food database, but also helps the coach in charge check daily to keep the dietary control.

- If Noom is used in the process of dietary adaptation after surgery for gastric cancer patients, it is expected that balanced nutritional intake will be achieved through appropriate dietary disorders. In addition, by providing information on gastrectomy syndrome, it is possible to reduce and prevent gastrectomy syndrome.

- Also, as Noom actively encourages exercise therapy, the patient is motivated for proper exercise.

- This is expected to reduce the patient's symptoms and improve the quality of life.

(4) **Previous literature review**

- In the past literature, the results that weight control and metabolic syndrome control were significantly improved when using a smartphone app were published in several papers.

- There have also been studies showing that breast cancer survivors, which are highly related to obesity, lose weight, increase vegetable intake, and increase exercise volume when nutritional control using a smartphone app is used.

- Regarding stomach cancer, a study on improving lung capacity with a mobile self-monitoring tool has been published, and studies related to nutrition or diet have only been introduced with protocols so far.


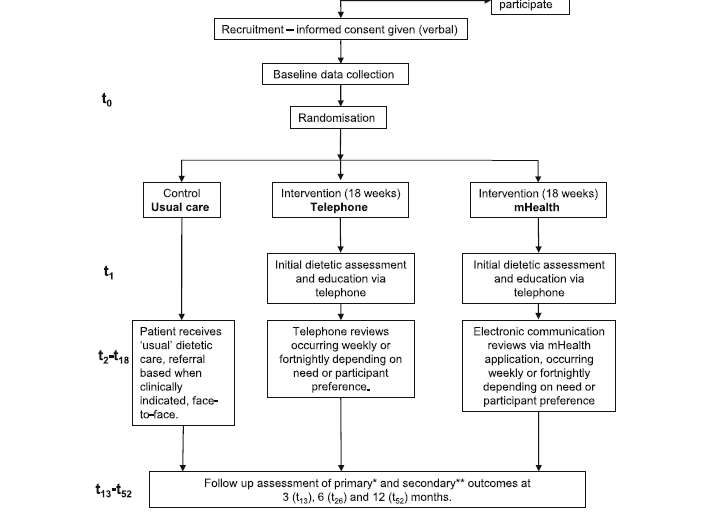


<Hanna et la, Effect of early and intensive nutrition care, delivered via telephone or mobile application, on qaulity of life in people with upper gastrointestinal cancer: study protocol of a randomized controlled trial, BMC Cancer, 2018>

**(5) Results of pilot study**

**- Research title**

: Investigation of the effectiveness of QOL improvement using a smartphone app-based human coaching program in patients who underwent gastrectomy for stage 1 gastric cancer.

**- Research content**

√ Target patient: Patients who underwent gastrectomy for stage 1 gastric cancer

√ Human Coaching Program Usage Period: 3 months from discharge date

√ Study method: Before discharge, the patient installs an app for the human coaching program on his/her smartphone and is informed of how to use it. After discharge, you receive individual coaching on how to manage your health after gastrectomy such as weight, diet, and exercise using a smartphone app.

√ Outcomes:

- EORTC QLQ questionnaire performing at 1 and 3 months postoperatively.
- Satisfaction with using the app
- Weight, BMI, body component
- Nutritional parameters (Hb, protein, albumin, cholesterol)

**- Patient enrollment**

√ Period of enrollment: 2019.9.19. ~ 2019.11.6

√ Number of enrolled patients (Actual/Planned): 20/20 (100%)

√ Number of drop-out: 6/20 (30%)

√ Reason of drop-out:

- Diagnosed as stage 2 or higher in the final pathology examination (n=4)

- Refusal (n=2)

**- Results**

1) Eating restriction score of EORTC QLQ-STO22

-Preoperative, median value: 6.7 (IQR 6.7, 13.3)

-Postoperative 1-month, median value: 30.0 (IQR 13.3, 56.7)

-Postoperative 3-month, median value: 20.0 (IQR 3.3, 26.7)

2) Satisfaction (n=13)

: The use of the app helped adapt to the diet and the overall satisfaction was very high.

-Ease of use of the app – Strongly yes (9), yes (3), no (1)

-Ease of understanding of the information provided - Strongly yes (8), yes (4)

-Usability of using the app - Strongly yes (12), yes (1)

-Appropriateness of time spent in app - Strongly yes (8), yes (5)

-Overall satisfaction: Strongly yes (11), yes (2)

(6) **Benefit**

- Evaluation of whether dietary management using a smartphone app contributes to improving the patient's quality of life in patients undergoing gastrectomy

- Development of coaching materials for patients undergoing gastrectomy

**2. Study Objectives**

**(1) Primary endpoint:**

- Analysis of the effect of using a smartphone app-based human coaching program on the patient's symptoms and quality of life in patients who underwent gastrectomy for stage 1 gastric cancer

- Comparison of food restriction scale scores of EORTC QLQ C30 and STO22 at 1 month after surgery between the patients who used smartphone app and those who received nutrition counseling

**(2) Secondary endpoints:**

- Food restriction scale score at 3, 6, 12 months after operation

- Meal amount (total calories, nutrient amount)

- Body component (body weight, body mass index, muscle mass, body fat percentage)

- Nutritional outcomes (hemoglobin, protein, albumin, cholesterol)

**3. Study Design**

(1) Study design: single center, prospective, randomization

(2) Research participation period: One year after gastrectomy for early gastric cancer

(3) Data collection period: IRB approval day – 2021. 12. 31

(4) Overall study period: IRB approval day – 2022. 6. 30.

**4. Study Population**

**(1) Target disease**

Stage I gastric cancer

**(2) Inclusion criteria**

1. Patients over 19 years of age

2. Patients diagnosed with stage 1 gastric cancer before surgery

(Early gastric cancer at preoperative endoscopy or stage I at CT scan)

3, Patients scheduled to undergo distal or total gastrectomy

4. Patients who are able to use the smartphone app or a care giver living with him can use the app to actively intervene in the patient's life.

5. Patients who have written research consent

**(3) Exclusion criteria**

1. Vulnerable patients (pregnancy, a person who lacks the ability to speak)

2. Patients who cannot use the smartphone app or the active intervention of a care-giver is difficult

**(4) Drop-out criteria**

1. Patients who have not undergone distal gastrectomy or total gastrectomy

1. The pathology test result is stage II or higher after gastrectomy
2. Patients who need adjuvant chemotherapy after gastrectomy

4. The hospital stay exceeds 3 weeks due to complications

5. Patients request withdrawal of participation

6. Patients having difficulty in collecting data suitable for the purpose of the study because they have not visited at all 1, 3, 6, and 12 months

1. **Study diagram**

|  | Informed consent | |  |
| --- | --- | --- | --- |
|  |  |  |  |
|  | Screening | |  |
|  |  |  |  |
|  | Gastrectomy | |  |
|  |  |  |  |
|  | Follow-up at 1, 3, 6, 12 months after operation | |  |

| **Data** | **Preop** | **Op** | **POD5** | **POD1m** | **POD3m** | **POD6m** | **POD12m** |
| --- | --- | --- | --- | --- | --- | --- | --- |
| age, sex, height, past medical history, smoking, alcohol, concomitant medications | O |  |  |  |  |  |  |
| operating day, operation method |  | O |  |  |  |  |  |
| pathological results of gastric cancer, postoperative complications |  |  |  | O |  |  |  |
| body weight | O |  | O | O | O | O | O |
| body component such as muscle mass measured with a body composition analyzer | O |  |  | O | O | O | O |
| EORTC QLQ C30, STO22 | O* |  |  | O | O | O | O |
| Hb, Protein, Albumin, Cholesterol | O |  | O |  | O | O | O |

**6. Patient Recruitment & Registration**

(1) **Research subject recruitment method and procedure**

- For patients who have been diagnosed with stage 1 gastric cancer and are expected to undergo gastrectomy, the attending physician directly explains and obtains consent to patients who meet the selection criteria and exclusion criteria.

- The consent process for participation in the study is to be made voluntarily after the consent is explained in detail to the subject, and the subject has sufficient time to think and ask questions.

- A copy of the consent form signed by the study coordinator is provided to the subject and the original is kept.

(2) **Number of research subjects**

- In the results of the pilot study, the eating restriction score of EORTC QLQ-STO22 that was answered at 1 month postoperatively was 30 points. The drop-out rate of the entire study was 30%. Based on this, the following hypothesis was established.

- Among the EORTC STO22 items that patients in the mHealth group responded to 1 month after surgery, the score for the eating restriction item was expected to be 30±10 (mean±standard deviation). The item score for the control group was expected to be 35 points.

- alpha value: 0.05

- power: 80%

→ sample size was calculated as 65 cases in each group.

→ Considering 30% of drop-out rate, a total of 180 cases (90 cases in each group) is needed.

- If drop-outs are more than expected, the number of sample size can be adjusted through statistical advice.

**7. Study Assessment & Efficacy**

**(1) Data collection**

○ Demography: age, sex, name initial

○ Anthropometric information: height, body weight, body component such as muscle mass measured with a body composition analyzer

○ Past medical history: past medical history, smoking, alcohol, concomitant medications

○ Diagnostic, pathological data: operating day, operation method, pathological results of gastric cancer, postoperative complications

○ Laboratory findings: Hemoglobin, Protein, Albumin, Cholesterol

○ Questionnaires: EORTC QLQ C30, STO22

**(2) Evaluation**

Between the mHealth and face-to-face counseling groups

- Comparison of scores of EORTC QLQ-C30 and STO22 at 1, 3, 6, and 12 months postoperatively.

- Comparison of meal amount at 1, 3, 6, and 12 months postoperatively.

- Comparison of body component (BMI, muscle mass, etc) at 1, 3, 6, and 12 months postoperatively.

- Comparison of nutritional outcomes (hemoglobin, albumin, protein, cholesterol) at 1, 3, 6, and 12 months postoperatively

**(3) Evaluation method**

- PI evaluates the completion of the performance.

- When analyzing, seek advice from the statistics team if necessary.

**(4) Evaluation time point:**

- Study completion day (2022. 6. 30.)

**(5) Outcome parameters**

- Comparison of food restriction scale score of EORTC QLQ C30, STO22:

Whether there is a significant difference in the mean value is evaluated by the p-value of the t-test. Since the 1st month questionnaire is the primary endpoint, it is evaluated individually and the significance of the mixed model's p-value is evaluated to see if there are overall differences in the values ​​measured several times.

- Inbody values ​​such as meal volume, BMI, muscle mass, and blood tests were compared with the average value at 3 months, and the significance was evaluated by the p-value of the mixed model to see if there was a difference overall.

**8. Study method**

**(1) Study method**

- Patients who are scheduled to undergo gastrectomy for stage 1 gastric cancer are randomly assigned either to the mHealth group or the face-to-face counseling group. Patients in the mHealth group (N=90) will use mobile application for 3 months after discharge and those who in the face-to-face counseling group (N=90) will receive general nutrition counseling at 1 month and 3 months after gastrectomy.


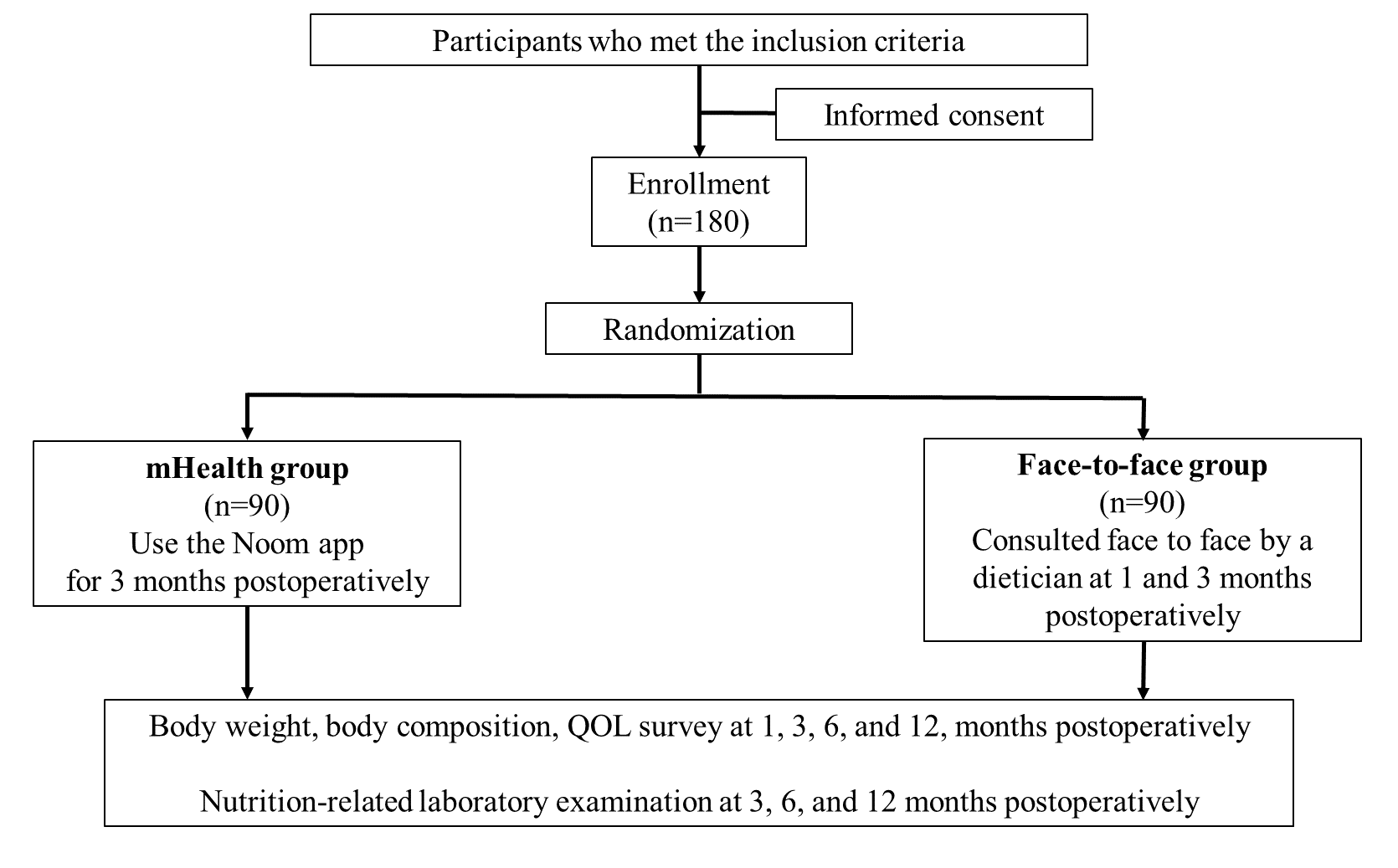


- Patients will receives nutritional counseling at 1 month (30 days) and 3 months (90 days) after gastrectomy by randomization. The allowable date of visit to the hospital is 1 month before and after 1, 3, 6, 12 months after surgery. If the patient does not visit the hospital and cannot be contacted, the study coordinator attempts to contact the hospital by phone or text once a week for a month. Subject will be eliminated from the study if he did not visit the hospital at all the follow-up period.

**(2) Schedule**

| List | Screening | Follow-up | Close |
| --- | --- | --- | --- |
| Check inclusion/exclusion criteria | X |  |  |
| Demography | X |  |  |
| Diagnosis, pathological results, complications | X | X |  |
| Body component |  | X |  |
| QoL questionnaire |  | X |  |
| Nutritional parameters |  | X |  |
| Use of smartphone app. |  | X |  |
| Report |  |  | X |

(3) **Study period**

- Research participation period: One year after gastrectomy for early gastric cancer

- Data collection period: IRB approval day – 2021. 12. 31

- Overall study period: IRB approval day – 2022. 6. 30.

**9. Statistical Methods**

(1) **Randomization**

- Randomization generally performs permutated mixed block randomization, and is generated in a 1:1 ratio using 2 and 4 mixed block blocks. Using the nQuery Program, a randomly assigned table is created according to the stratification factor and notified through a web-based clinical research management system.

- Stratification factors: extent of gastrectomy (distal vs. total gastrectomy)

(2) **Statistical analyses**

- Patient's clinical characteristics are expressed as mean ± standard deviation or ratio using descriptive statistics.

- Significance was evaluated by t-test for continuous variables and Chi-square test for discontinuous variables.

- For the values ​​repeatedly measured at time intervals, the difference between the two groups was compared using a mixed effect model.

**10. Interim analysis**

Not applicable

**11. Safety**

(1) This study is conducted through the use of smartphone apps and questionnaires of the subjects, and safety issues are not applicable.

(2) Adverse reactions are not collected because there are no adverse reactions in the use of smartphone apps or surveys.

**12. Ethical Considerations**

**(1)** Protection measures for vulnerable research subjects ☑ Not applicable

**(2)** How to use personal information including personally identifiable information

- Personal identifiable information: hospital registration number, name, telephone number, birth year

- Personal information: age, sex, height, weight, past medical history, smoking status, alcohol consumption, concomitant medication use, operation date, surgical method, gastric cancer pathology result, postoperative complications, BMI, body composition analysis and body measurement results, Hb, protein, albumin, cholesterol

- Purpose of using personal information, including personally identifiable information:

Search of medical record, smartphone app use and human coaching

- Use period of personal information including personally identifiable information:

- Stored for 3 years after the end of the study

- When a participant requests deletion of personal information, it will be deleted immediately

**(3)** Personal Information Protection Plan

- Subjects have the right to refuse consent to collection and use of personal information, and participation in this study is restricted if consent is rejected (Article 15 of the Personal Information Protection Act, collection and use of personal information), and minimum personal information necessary for the purpose of the study (Article 16 of the Personal Information Protection Act, Restriction on Collection of Personal Information). Records related to personal information will be kept for 3 years from the time the research is completed in accordance with Article 15 of the Enforcement Rule of the 「Life Ethics and Safety Act」. This past document will be destroyed (Article 21 of the Personal Information Protection Act, destruction of personal information).

- Disposal plan for data after research is completed: Research data including personal information will be disposed of as follows in accordance with Article 16 of the Enforcement Decree of the Personal Information Protection Act after the storage period.

- Information stored in the form of electronic files is permanently deleted in a way that cannot be restored through initialization or overwriting to prevent data from being restored.

- Written printed materials are completely destroyed through a shredder.

- Protection methods for research subjects and to ensure confidentiality of personal information

(1) Only the minimum personal information necessary for the purpose of this study will be collected, and the collected personal identification information and personal information will not be leaked or shared with others other than the responsible researcher and researcher

(2) Data will be managed by assigning a research ID, and the information collected for research will be stored as an electronic file with a password in a locked laboratory. Only PI and researchers can access the research file.

(3) It will be permanently deleted by PI after storage for 3 years after the end of the study.

(4) Research-related support

- Grant 1910242 of the National Cancer Center, Republic of Korea

(5) Support matters for research subjects

- Free use of smartphone app (Noom) for 3 months

- Nutrition education at 1 month and 3 months after surgery

(6) Additional costs incurred when a research subject participates in the research:

: Not applicable

**(7)** Data management plan

- Data is managed by assigning a research ID, and information collected for research is stored in an electronic file with a password in a locked laboratory.

- Research files can only be accessed by PI and researchers, and collected documents are kept in a lockable drawer to limit access.

- The collected data is stored for 3 years after the end of the study and then permanently disposed of by the lead researcher.

- The identifier code associated with the subject's personal information is restricted by PI by storing it in a lockable chest of drawers.

**13. References**

1. Lin PH, Grambow S, Intille S, Gallis JA, Lazenka T, Bosworth H, et al. The Association Between Engagement and Weight Loss Through Personal Coaching and Cell Phone Interventions in Young Adults: Randomized Controlled Trial. JMIR Mhealth Uhealth. 2018 Oct 18;6(10):e10471.

2. Tanaka K, Sasai H, Wakaba K, Murakami S, Ueda M, Yamagata F, Sawada M, Takekoshi K. Professional dietary coaching within a group chat using a smartphone application for weight loss: a randomized controlled trial. J Multidiscip Healthc. 2018 Jul 16;11:339-347.

3. Timpel P, Cesena FHY, da Silva Costa C, Soldatelli MD, Gois E Jr, Castrillon E, et al. Efficacy of gamification-based smartphone application for weight loss in overweight and obese adolescents: study protocol for a phase II randomized controlled trial. Ther Adv Endocrinol Metab. 2018 Jun;9(6):167-176.

4. Morawski K, Ghazinouri R, Krumme A, Lauffenburger JC, Lu Z, Durfee E, et al. Association of a Smartphone Application With Medication Adherence and Blood Pressure Control: The MedISAFE-BP Randomized Clinical Trial. JAMA Intern Med. 2018 Jun 1;178(6):802-809.

5. M Quintiliani L, Mann DM, Puputti M, Quinn E, Bowen DJ. Pilot and Feasibility Test of a Mobile Health-Supported Behavioral Counseling Intervention for Weight Management Among Breast Cancer Survivors. JMIR Cancer. 2016 May 9;2(1):e4.

6. Wu JM, Yu HJ, Ho TW, Su XY, Lin MT, Lai F. Tablet PC-enabled application intervention for patients with gastric cancer undergoing gastrectomy. Comput Methods Programs Biomed. 2015 Apr;119(2):101-9.
